# Breaking the adherence barrier: an information–motivation–behavioral skills model analysis of self-efficacy in stroke survivors’ home-based exercise

**DOI:** 10.3389/fpubh.2026.1747370

**Published:** 2026-04-16

**Authors:** Wenbo Li, Qiujie Li

**Affiliations:** Department of Clinical Nursing Education, The Second Affiliated Hospital of Harbin Medical University, Harbin, China

**Keywords:** adherence, home-based exercise, IMB model, ischemic stroke, rehabilitation, self-efficacy, SEM

## Abstract

**Background:**

Adherence to home-based exercise is essential for functional recovery after stroke, yet non-adherence is common and poorly explained. The Information–Motivation–Behavioral Skills (IMB) model has been widely applied in health behavior research but rarely tested in stroke rehabilitation. This study examined behavioral determinants of exercise adherence and tested the mediating role of self-efficacy based on the IMB framework.

**Methods:**

A cross-sectional survey was conducted among 549 ischemic stroke survivors with limb dysfunction recruited from rehabilitation clinics in Harbin, China. Participants completed validated measures of exercise adherence, stroke-related knowledge, motivation for exercise, perceived social support, and self-efficacy. Structural equation modeling (SEM) was used to test hypothesized IMB pathways while controlling for demographic and clinical covariates.

**Results:**

The final model demonstrated good fit (*χ^2^*/df = 2.675, RMSEA = 0.055, CFI = 0.949). Self-efficacy had the strongest direct effect on adherence (*β* = 0.37, *p* < 0.001) and significantly mediated the effects of stroke knowledge (indirect effect = 0.119, *p* < 0.001), personal motivation (0.078, *p* < 0.001), and social support (0.056, *p* < 0.001). Motivation also had a direct effect on adherence (*β* = 0.25, *p* < 0.001), while knowledge and social support influenced adherence only indirectly via self-efficacy.

**Conclusion:**

Self-efficacy is a central behavioral mechanism underlying home exercise adherence after stroke. The IMB framework offers a useful foundation for designing adherence-enhancing interventions that build confidence, reinforce progressive mastery, and integrate social support in rehabilitation planning.

## Introduction

Stroke remains a leading cause of long-term disability worldwide, with ischemic stroke accounting for more than 70% of all cases (1). Despite advances in acute treatment, many stroke survivors experience persistent motor impairment requiring prolonged rehabilitation (2). Home-based exercise is a central component of post-stroke rehabilitation and is recommended in international stroke guidelines due to its proven benefits in improving functional independence, balance, and quality of life (3, 4). However, poor adherence to prescribed home exercise programs is common, with reported adherence rates remaining below 50% (5). Low engagement in rehabilitation activities substantially limits recovery potential and contributes to long-term disability, increased risk of recurrent stroke, and greater healthcare burden.

Understanding why stroke survivors fail to adhere to home exercise remains a major challenge. Previous studies have identified several influencing factors, such as age, depression, physical limitations, and social support (6, 7). However, most existing research has been descriptive and lacked a theoretical framework to explain how psychological and behavioral factors interact to affect adherence (8). Moreover, many studies focus on hospital-based rehabilitation or supervised exercise interventions, while little is known about behavioral determinants of home-based exercise adherence, which relies heavily on patient self-management (9, 10). Without a behavioral explanation, interventions often emphasize knowledge delivery alone and fail to produce sustained improvements in exercise behavior (11).

The Information–Motivation–Behavioral Skills (IMB) model was developed by Fisher et al. in the 1990s (12). Originally developed to explain health-related adherence and preventive behaviors, the model proposes that behavior is determined by three key constructs: information, motivation, and behavioral skills. Information refers to accurate and relevant knowledge about the health condition and the required behavior, such as understanding the purpose of rehabilitation exercise, exercise precautions, and the consequences of poor adherence. Motivation includes both personal motivation, such as beliefs, attitudes, and willingness to perform exercise, and social motivation, such as encouragement and support from family members, caregivers, and healthcare professionals. Behavioral skills refer to the practical ability and perceived confidence needed to perform the target behavior consistently in everyday life, with self-efficacy considered a central component. According to the IMB model, information and motivation may influence behavior directly, but they often exert their effects indirectly through behavioral skills. Thus, even when patients understand rehabilitation instructions and recognize their importance, adherence may remain suboptimal if they do not feel capable of carrying out exercise independently and overcoming barriers at home. This framework is particularly relevant to stroke rehabilitation, where home-based exercise requires not only knowledge and motivation but also confidence, problem-solving ability, and sustained self-management under conditions of physical and psychological limitation.

This relevance is further supported by previous studies showing that the IMB model has been successfully applied in a range of chronic disease contexts, including medication adherence, diabetes self-management, and cardiovascular rehabilitation (13, 14). Compared with purely descriptive approaches, this model is particularly useful because it not only identifies relevant determinants of behavior but also explains how these determinants may operate together. In stroke rehabilitation, home-based exercise adherence is likely shaped by whether patients possess sufficient rehabilitation knowledge, maintain motivation to continue exercise, receive adequate social support, and believe they can successfully perform exercise tasks despite ongoing limitations. Nevertheless, the application of the IMB model to stroke survivors’ home-based exercise remains limited, and few studies have used structural equation modeling to examine the pathways linking these constructs to adherence behavior (15). Further investigation is therefore needed to clarify these relationships in stroke populations.

Therefore, this study aimed to apply the IMB model to explain home-based exercise adherence among ischemic stroke survivors. We tested a structural equation model to: (1) examine associations between stroke-related knowledge, exercise motivation, social support, self-efficacy, and adherence; and (2) evaluate whether self-efficacy mediates the effects of information, motivation, and social support on adherence. By clarifying behavioral pathways, this study provides a theory-driven foundation for developing targeted interventions to improve rehabilitation engagement after stroke.

## Methods

### Study design and ethics

This cross-sectional study was conducted in Harbin, China, between May and July 2025. The protocol was approved by the Ethics Committee of the Second Affiliated Hospital of Harbin Medical University (Approval No. KY2025-169). All participants provided written informed consent prior to enrollment.

### Participants

Using convenience sampling, 549 patients with ischemic stroke and limb dysfunction were recruited from rehabilitation clinics. Inclusion criteria were: (1) age ≥18 years; (2) ischemic stroke confirmed by CT or MRI; (3) clinically stable recovery phase with motor impairment; and (4) ability to complete the survey independently or with assistance. Exclusion criteria included: (1) severe comorbid illness (e.g., advanced cardiac, pulmonary, or renal disease, or malignancy) precluding participation; (2) significant cognitive impairment or psychiatric disorder; and (3) severe communication or reading difficulties.

### Sample size

Sample size was estimated using G*Power 3.1.9.6 for multiple regression, assuming a medium effect size (*f^2^* = 0.15), *α* = 0.05, power = 0.95, and 24 predictors. The minimum required sample was 238. Allowing for a 20% invalid response rate, at least 286 participants were needed. To ensure stability for structural equation modeling, 600 potentially eligible patients were approached. After eligibility assessment and informed consent, the questionnaire link or QR code was provided to those who agreed to participate. Instead, 549 valid questionnaires were included in the final analysis.

## Measures

### Descriptive information form

The descriptive information form was developed by the research team after a targeted literature review. It comprised 11 self-reported items capturing demographic characteristics (gender, age, education level, marital status, pre-illness employment status, monthly income, living arrangement, and primary caregiver) and disease-related variables (presence of comorbid chronic diseases, number of strokes, and participation in discharge rehabilitation instruction).

### Exercise adherence questionnaire (EAQ)

The EAQ, developed by Lin Beilei et al. in 2013 (16), measures adherence to functional exercise during stroke rehabilitation. It contains 14 items across three domains—participation in exercise, monitoring of exercise outcomes, and seeking exercise guidance—rated on a 4-point Likert scale from 1 (“Not at all possible”) to 4 (“Completely possible”). Total scores range from 14 to 56, with higher scores indicating better adherence. Internal consistency in this study was good (Cronbach’s *α* = 0.875).

### Stroke knowledge questionnaire (SKQ)

The SKQ, developed by Yao Qiping (17), assesses stroke-related knowledge across six domains: emergency response, warning signs, risk factors, healthy behaviors, rehabilitation, and medication safety. The 40 items are scored dichotomously (1 = correct; 0 = incorrect/“do not know”), yielding a total score of 0–40; higher scores reflect greater knowledge. Internal consistency in this study was good (Cronbach’s *α* = 0.862).

### Behavioral regulation in exercise Questionnaire-3 (BREQ-3)

The BREQ-3 comprises 24 items covering six forms of motivation—intrinsic, integrated, identified, introjected, external, and amotivation (four items each) (18). Items are rated on a 5-point Likert scale from 0 (“Not true at all”) to 4 (“Very true”). Dimension means are computed, and the Relative Autonomy Index (RAI) is calculated as: RAI = (3 × intrinsic) + (2 × integrated) + identified − introjected − (2 × external) − (3 × amotivation). Higher RAI values indicate more autonomous motivation. The Chinese version translated by Wen Fan et al. has shown good psychometrics in university students (Cronbach’s *α* = 0.828) (17). In this study, internal consistency was acceptable (Cronbach’s *α* = 0.721).

### General self-efficacy scale (GSES)

The GSES evaluates confidence in managing daily demands and challenges (19). It includes 10 items rated on a 4-point Likert scale from 1 (“Not at all true”) to 4 (“Exactly true”), producing a total score of 10–40; higher scores indicate stronger self-efficacy. Internal consistency in this study was good (Cronbach’s *α* = 0.810).

### Perceived social support scale (PSSS)

The PSSS, developed by Zimet et al. (20), measures perceived social support across three subscales—family (items 3, 4, 8, 11), friends (items 6, 7, 9, 12), and significant others/other support (items 1, 2, 5, 10). Items are rated on a 7-point Likert scale from 1 (“Very strongly disagree”) to 7 (“Very strongly agree”), yielding total scores of 12–84; higher scores denote greater perceived support. The original scale demonstrated high internal consistency (Cronbach’s *α* = 0.921); in this study, Cronbach’s α was 0.888.

### Data collection

Data were collected using the Wenjuanxing online survey platform^1^. Participants accessed the questionnaire via Quick Response code or link distributed on WeChat. Prior to participation, study aims, procedures, and rights were explained by trained staff. Confidentiality and voluntary participation were assured. To safeguard confidentiality, the questionnaire did not request direct personal identifiers. Informed consent records were stored separately from survey responses. The dataset used for analysis was de-identified prior to export. All study personnel signed confidentiality undertakings, and data retention and destruction followed institutional policy. Only aggregate results are reported, and no individual-level information is disclosed to third parties.

### Statistical analysis

Analyses were performed using SPSS 27.0 and Amos 26.0 (IBM Corp., Armonk, NY, United States). Continuous variables were summarized as mean ± standard deviation (SD), and categorical variables as frequencies and percentages. Group differences in exercise adherence scores were examined using independent-samples t tests or one-way analysis of variance, as appropriate. Pearson correlation analysis was used to assess bivariate associations among stroke-related knowledge, exercise motivation, perceived social support, self-efficacy, and exercise adherence.

Structural equation modeling (SEM) was conducted in Amos 26.0 to test the hypothesized pathways of the IMB model. In the present study, stroke-related knowledge represented the information component, exercise motivation represented the motivation component, self-efficacy represented behavioral skills, and exercise adherence was specified as the behavioral outcome. Perceived social support was additionally included as a contextual factor relevant to motivation and behavioral performance in stroke rehabilitation. Based on the IMB framework, a hypothesized model was established in which information, motivation, and perceived social support were assumed to have direct effects on exercise adherence and indirect effects through self-efficacy. Paths from information, motivation, and perceived social support to self-efficacy were also specified.

The observed total scores of the SKQ, BREQ-3 Relative Autonomy Index, PSSS, GSES, and EAQ were entered into the model as manifest variables. Maximum likelihood estimation was used to estimate model parameters. Model fit was evaluated using multiple goodness-of-fit indices, including the chi-square to degrees of freedom ratio (*χ^2^*/df), root mean square error of approximation (RMSEA), comparative fit index (CFI), normed fit index (NFI), incremental fit index (IFI), goodness-of-fit index (GFI), and adjusted goodness-of-fit index (AGFI). Acceptable model fit was defined as *χ^2^*/df ≤ 3.0, RMSEA < 0.06, and CFI, NFI, IFI, GFI, and AGFI > 0.90. Model modification was performed with reference to modification indices and theoretical plausibility. Standardized path coefficients were used to interpret direct and indirect effects. A two-tailed *p* < 0.05 was considered statistically significant.

## Results

### Demographic characteristics

A total of 549 participants were included in the analysis. Women accounted for 52.2% of the sample. The largest age groups were 70–79 years (21.1%) and ≥80 years (21.7%). Nearly one-third (29.5%) had a college education or above, while 49.9% reported comorbid chronic conditions.

Exercise adherence differed significantly by gender (*p* = 0.023), with men scoring higher than women, and by marital status (*p* < 0.001), with unmarried individuals showing the highest adherence. Living arrangement was also associated with adherence (*p* = 0.001), with nursing home residents demonstrating better adherence than those living alone or with family. Patients with a single stroke episode had significantly higher adherence than those with multiple strokes (*p* < 0.001). No significant differences were observed across age, education level, employment status, income, or participation in discharge instruction. Full details are presented in Supplementary Table S1.

### Correlations among study variables

Exercise adherence was positively correlated with stroke-related knowledge (*r* = 0.287, *p* < 0.001), exercise motivation (*r* = 0.452, *p* < 0.001), self-efficacy (*r* = 0.539, *p* < 0.001), and perceived social support (*r* = 0.350, *p* < 0.001). Correlation coefficients for all variables are shown in Supplementary Table S2.

### Mediation analysis

Mediation tests indicated that self-efficacy mediated the effects of knowledge (indirect effect = 0.119, *p* < 0.001), personal motivation (0.078, *p* < 0.001), and social support (0.056, *p* < 0.001) on adherence (Table 1). These findings suggest that self-efficacy acts as a key psychological mechanism linking information, motivation, and social support to actual exercise behavior.

**Table 1:** Direct effect, indirect effect and total effect of model variables on exercise adherence.

Variable	The model			
	Estimate	SE	*p*-Value	95% CI [Lower, Upper]
Information → exercise adherence				
Direct effect	−	−	−	−
Indirect effect via self-efficacy	0.119	0.018	<0.001	[0.086, 0.158]
Total effect	0.119	0.018	<0.001	[0.086, 0.158]
Personal motivation → exercise adherence				
Direct effect	0.182	0.032	<0.001	[0.121, 0.245]
Indirect effect via self-efficacy	0.078	0.014	<0.001	[0.053, 0.109]
Total effect	0.259	0.031	<0.001	[0.197, 0.320]
Social support → exercise adherence				
Direct effect	−	−	−	−
Indirect effect via self-efficacy	0.056	0.011	<0.001	[0.037, 0.079]
Total effect	0.056	0.011	<0.001	[0.037, 0.079]
Self-efficacy → exercise adherence				
Direct effect	0.182	0.032	<0.001	[0.121, 0.245]
Total effect	0.182	0.032	<0.001	[0.121, 0.245]

### Model estimation

**Figure 1:**
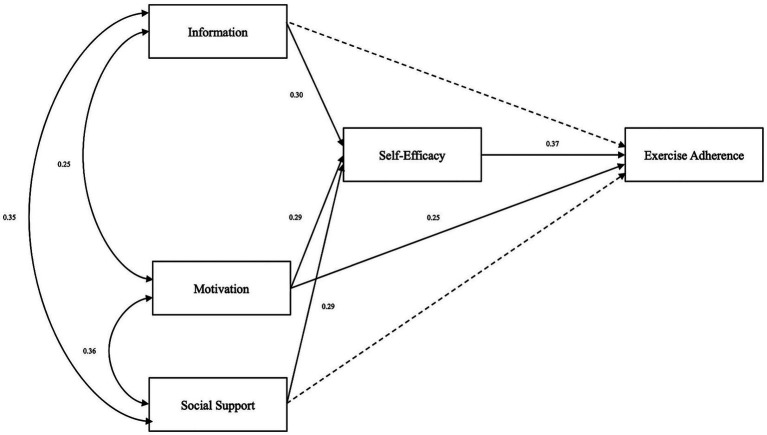
Structural equation model based on the IMB framework (*N* = 549). Values represent standardized path coefficients. All paths shown are statistically significant at *p* < 0.05. The model was tested with demographic and clinical variables including gender, marital status, living arrangement, and number of strokes as covariates, though these are not displayed for visual clarity.

The hypothesized IMB model demonstrated good overall fit to the data. Specifically, the fit indices were *χ^2^*/df = 2.675, RMSEA = 0.055, NFI = 0.922, IFI = 0.950, CFI = 0.949, GFI = 0.975 and AGFI = 0.952, all of which met or exceeded commonly accepted criteria for acceptable model fit (Supplementary Table S3). Path analysis results demonstrated that exercise motivation directly predicted adherence (*β* = 0.25, *p* < 0.05) and indirectly influenced adherence through self-efficacy (*β* = 0.29, *p* < 0.05). Knowledge had no direct effect but significantly influenced adherence via self-efficacy (*β* = 0.30, *p* < 0.05). Social support also showed no direct effect but significantly enhanced self-efficacy (*β* = 0.29, *p* < 0.05). Self-efficacy itself exerted the strongest direct effect on adherence (*β* = 0.37, *p* < 0.05). Additional correlations were observed among the IMB components: knowledge was positively correlated with both social support (*r* = 0.35, *p* < 0.05) and motivation (*r* = 0.25, *p* < 0.05), and social support correlated positively with motivation (*r* = 0.36, *p* < 0.05). Standardized path coefficients are presented in Supplementary Table S4, and the final structural model is shown in Figure 1.

## Discussion

This study applies the IMB framework to explain why stroke survivors fail to adhere to home-based exercise and demonstrates that self-efficacy is the central mechanism translating information, personal/social motivation, and support into actual exercise behavior. In our structural model, self-efficacy showed the strongest direct effect on adherence and fully mediated the effects of knowledge and social support, while motivation exerted both direct and indirect effects. These patterns clarify behavioral pathways underlying non-adherence after discharge and identify leverage points for intervention.

### Model fit and robustness of the structural model

In addition to the theoretical interpretation of the IMB pathways, the adequacy of model fit is an important prerequisite for the credibility of SEM findings. In the present study, the structural model demonstrated satisfactory fit indices (*χ^2^*/df = 2.675, RMSEA = 0.055, NFI = 0.922, IFI = 0.950, CFI = 0.949, GFI = 0.975, AGFI = 0.952). According to commonly accepted SEM evaluation criteria, a *χ^2^*/df value below 3 indicates acceptable model parsimony, while RMSEA values below 0.06 reflect good approximation of the model to the observed data. Similarly, incremental fit indices such as CFI, NFI, and IFI greater than 0.90 suggest that the proposed model explains the covariance structure of the data substantially better than a null model. The high GFI and AGFI values further indicate that the hypothesized structure adequately reproduces the observed variance–covariance matrix.

These results suggest that the IMB-based structural model provides a statistically sound representation of the relationships among knowledge, motivation, social support, self-efficacy, and exercise adherence in stroke survivors. Comparable model fit has been reported in previous IMB-based SEM studies examining health behaviors such as medication adherence and self-management in chronic disease populations, where CFI values typically ranged from 0.90 to 0.95 and RMSEA values from 0.04 to 0.07 (21–24). Therefore, the model fit indices observed in the present study fall within the recommended ranges and are consistent with prior IMB applications, further supporting the validity and robustness of the proposed behavioral pathways.

### Self-efficacy as a key mediator

Consistent with previous research, self-efficacy emerged as the strongest predictor of adherence. The standardized direct path from self-efficacy to exercise adherence ( β=0.37 ) was larger than that of motivation ( β=0.25 ) and exceeded the direct effects of the other IMB-related constructs, highlighting self-efficacy as the most proximal behavioral determinant in this framework. In practical terms, this coefficient indicates a moderate and clinically meaningful association: stroke survivors with higher confidence in their ability to perform prescribed exercises were substantially more likely to sustain home-based rehabilitation behavior.

This finding is consistent with IMB-based studies in stroke self-management and other chronic disease populations, where behavioral skills or self-efficacy often show the strongest direct association with adherence-related outcomes. Zhang et al. synthesized patient, caregiver, and clinician perspectives and highlighted low exercise self-efficacy and depressive mood as core barriers, while tailored prescriptions and monitoring/feedback were seen as enablers—maps neatly onto IMB’s “skills” construct (self-efficacy) and “information” provision (25). Kim et al. (26) modeled stroke self-management using IMB and likewise found behavioral skills/self-efficacy to be proximal drivers. Beyond stroke, IMB trials and SEM studies in diabetes and epilepsy consistently show that knowledge and motivation improve adherence chiefly by elevating self-efficacy (7, 13, 27). Together with the present findings, these studies strengthen the case that building mastery experiences, offering timely positive feedback, and structuring practice should be central to adherence interventions (18, 28).

### The dual role of motivation

Our model indicates that exercise motivation not only correlates with adherence but exerts a direct effect and an indirect effect via self-efficacy. This “dual pathway” mirrors IMB applications to heart-healthy lifestyles in which intrinsic motivation showed a strong direct relationship with behavior and also fed into skills/self-efficacy (29). Longitudinal evidence further suggests that higher baseline motivational readiness predicts membership in high or improving-adherence trajectories over time (27). Mixed-methods work in community stroke survivors similarly points to attitudes and sustained routines as adherence engines, especially when paired with simplified, progressive tasks and supportive environments (9). Clinically, this implies our programs should go beyond didactics to activate autonomous motivation—for example, via motivational interviewing, shared goal-setting, and visualizing recovery progress—while simultaneously scaffolding skill acquisition.

### Translating knowledge and social support into adherence

Although knowledge and social support correlated with adherence, their effects were indirect through self-efficacy in our SEM. This pattern echoes IMB-guided work across conditions showing that education and support are necessary but insufficient without concomitant gains in confidence and skills (26, 30). Qualitative (25) and mixed-methods findings (9) similarly underscore that “knowing what to do” must be paired with “believing I can do it in my context.” Importantly, the quality of feedback matters: a 6-month digital-health study in older adult stroke patients showed that high-value, personalized feedback improved adherence, whereas over-frequent, low-value nudges slightly undermined it (31). This nuance aligns with our mediation results: information is most impactful when it builds mastery rather than merely repeating instructions. Social support likely strengthens self-efficacy through emotional encouragement, verbal persuasion, and instrumental help, again fitting the indirect pathway we observed.

### Integrative significance of the IMB model

This study extends the IMB model to stroke rehabilitation, a context characterized by physical disability, cognitive challenges, and fluctuating psychological states. The observed positive correlations among knowledge, motivation, and social support reflect the synergistic nature of IMB components. By clarifying the behavioral mechanisms underlying home-based exercise, our study provides a theory-driven foundation for designing comprehensive interventions that simultaneously address information gaps, motivation, and self-efficacy. Such integrative strategies may prove more effective than single-component approaches (8, 14, 32).

### Contextual considerations and generalizability

It is also important to consider the geographic context of the study when interpreting the findings. All participants were recruited from rehabilitation clinics in Harbin, Heilongjiang Province, which represents a specific healthcare and sociocultural environment in northeastern China. Regional differences in healthcare infrastructure, accessibility of community rehabilitation services, socioeconomic conditions, and family caregiving patterns may influence patients’ motivation, perceived social support, and engagement in home-based exercise. Therefore, while the IMB-based behavioral relationships identified in this study are theoretically grounded, their strength and manifestation may differ in other geographic or healthcare contexts. Future studies should validate the model in multicenter samples across different regions and healthcare systems to further examine its generalizability.

#### Clinical implications

This study indicates that enhancing self-efficacy is central to improving adherence to home-based exercise among stroke survivors. In clinical practice, health education or social support alone is often insufficient; patients require stepwise exercise prescriptions and timely positive feedback to build confidence and reinforce their sense of competence. At the same time, individualized goal-setting and motivational interviewing can foster stronger intrinsic motivation, helping patients maintain long-term commitment to rehabilitation. It is noteworthy that lower adherence was observed among women, divorced or widowed individuals, community-dwelling survivors, and those with recurrent strokes, suggesting that these groups warrant additional clinical attention, including more frequent follow-ups, caregiver involvement, or simple monitoring tools, to better support their recovery.

#### Strengths and limitations

This theory-driven study applied an IMB-based mechanistic framework to a large clinical sample using structural equation modeling, demonstrating good overall model fit and allowing for the quantification of indirect effects while adjusting for key sociodemographic and clinical factors. In addition, validated instruments were used to comprehensively capture the core IMB constructs, including information, motivation, behavioral skills (self-efficacy), and exercise adherence. Several limitations should also be acknowledged. First, the cross-sectional design and reliance on self-reported measures limit causal inference and may introduce common-method bias. Second, all participants were recruited from a single city, which may restrict the external validity and generalizability of the findings to other regions or healthcare settings. Future multicenter studies involving more diverse populations are needed to further validate the robustness of the IMB-based behavioral pathways identified in this study. Third, all variables were assessed at a single time point, preventing the examination of temporal relationships among variables. Longitudinal or experimental studies are therefore required to establish causal ordering and evaluate the durability of the observed associations. In addition, detailed information on the interval between stroke onset and study recruitment was not collected. Because patients at different stages of post-stroke recovery may differ in functional status, rehabilitation needs, self-efficacy, and exercise adherence, the potential influence of recovery stage on the stability of the findings could not be fully assessed. Future studies should record this interval more precisely and consider stratified analyses or statistical adjustments based on rehabilitation stage. Moreover, because the study focused on home-based exercise adherence after discharge, WeChat-based follow-up via the Wenjuanxing platform was used as a practical method for data collection in the home setting. However, this approach may have preferentially included patients with smartphone access and sufficient digital literacy, thereby introducing potential selection bias. Finally, detailed individual-level information on non-respondents and incomplete respondents was not systematically collected, meaning that potential non-response bias cannot be fully excluded.

## Conclusion

This study, guided by the Information-Motivation-Behavioral Skills model, identified self-efficacy as the central determinant of home exercise adherence among stroke survivors. Knowledge and social support exerted their effects primarily through enhanced self-efficacy, while motivation influenced adherence both directly and indirectly. These findings suggest that education or support alone is insufficient to sustain behavioral change; instead, clinical interventions should prioritize building patients’ confidence and competence, with particular attention to high-risk groups, in order to achieve more sustained and effective rehabilitation outcomes.

## Data Availability

The raw data supporting the conclusions of this article will be made available by the authors, without undue reservation.

## Footnotes

1
https://www.wjx.cn
