# Supplementary material for: Breaking the adherence barrier: an information–motivation–behavioral skills model analysis of self-efficacy in stroke survivors’ home-based exercise
Source: Front Public Health. 2026 Apr 16;14:1747370. doi: 10.3389/fpubh.2026.1747370 (PMC13131255; doi:10.3389/fpubh.2026.1747370)
Supplement: Supplementary file 1 [file Table_1.docx]

**Supplementary Table 1** Participants' characteristics and the median of the patient exercise adherence (n = 549).

| Socio-demographics | Number of participants N (row%) | Patient exercise adherence | |
| --- | --- | --- | --- |
|  |  | Mean ± SD | P |
| **Gender** |  |  | 0.023 |
| Male | 261 (47.5%) | 35.55 ± 7.75 |  |
| Female | 288 (52.2%) | 34.02 ± 8.00 |  |
| **Age** |  |  | 0.314 |
| <50 | 107 (19.5%) | 35.87 ± 7.15 |  |
| 50-59 | 107 (19.5%) | 35.24 ± 8.33 |  |
| 60-69 | 100 (18.2%) | 33.67 ± 8.96 |  |
| 70-79 | 116 (21.1%) | 34.36 ± 7.37 |  |
| ≥80 | 119 (21.7%) | 34.57 ± 7.72 |  |
| **Education level** |  |  | 0.064 |
| Primary school or below | 130 (23.7%) | 33.25 ± 8.61 |  |
| Junior high school | 117 (21.3%) | 34.66 ± 8.46 |  |
| Senior high school or technical secondary school | 140 (25.5%) | 35.12 ± 7.82 |  |
| College degree or above | 162 (29.5%) | 35.69 ± 6.82 |  |
| **Marital status** |  |  | < 0.001 |
| Never married | 82 (14.9%) | 39.06 ± 6.66 |  |
| Married | 245 (44.6%) | 36.02 ± 6.19 |  |
| Divorced/Widowed | 222 (40.4%) | 31.74 ± 8.89 |  |
| **Pre-illness employment status** |  |  | 0.755 |
| Unemployed | 166 (30.2%) | 34.36 ± 8.07 |  |
| Retired | 198 (36.1%) | 34.90 ± 7.52 |  |
| Employed | 185 (33.7%) | 34.92 ± 8.21 |  |
| **Monthly income** |  |  | 0.194 |
| <2,000 | 141 (25.7%) | 35.77 ± 7.31 |  |
| 2,000-5,000 | 225 (41.0%) | 34.50 ± 8.68 |  |
| >5,000 | 183 (33.3%) | 34.26 ± 7.32 |  |
| **Living arrangement** |  |  | 0.001 |
| Living alone | 97 (17.7%) | 34.21 ± 7.76 |  |
| Living with family | 174 (31.7%) | 33.99 ± 8.01 |  |
| Living with a caregiver/domestic helper | 152 (27.7%) | 33.87 ± 8.48 |  |
| Living in a nursing home/other institution | 126 (23.0%) | 37.27 ± 6.66 |  |
| **Primary caregiver** |  |  | 0.083 |
| Spouse | 151 (27.5%) | 35.63 ± 7.64 |  |
| Children | 151 (27.5%) | 34.77 ± 8.16 |  |
| Caregiver/domestic helper | 124 (22.6%) | 35.14 ± 7.65 |  |
| Others (e.g., parents, friends) | 123 (22.4%) | 33.24 ± 8.08 |  |
| **Presence of comorbid chronic diseases** |  |  | 0.355 |
| Yes | 272 (49.5%) | 35.06 ± 7.76 |  |
| No | 277 (50.5%) | 34.44 ± 8.06 |  |
| **Number of strokes** |  |  | < 0.001 |
| 1 | 159 (29.0%) | 36.66 ± 7.27 |  |
| 2 | 141 (25.7%) | 35.10 ± 8.00 |  |
| 3 | 142 (25.9%) | 33.97 ± 7.81 |  |
| ≥4 | 107 (19.5%) | 32.47 ± 8.24 |  |
| **Participation in discharge rehabilitation instruction** | |  | 0.995 |
| Participated | 267 (48.6%) | 34.75 ± 7.87 |  |
| Not participated | 282 (51.4%) | 34.74 ± 7.97 |  |

**Supplementary Table 2** Means, standard deviations, and correlation between exercise adherence, information, personal motivation, self-efficacy, and social support (n = 549).

|  | Mean ± SD | 1 | 2 | 3 | 4 | 5 | 6 | 7 |
| --- | --- | --- | --- | --- | --- | --- | --- | --- |
| 1.EAQ | 34.75 ± 7.91 | 1 |  |  |  |  |  |  |
| 2.SKQ | 28.04 ± 7.18 | 0.287** | 1 |  |  |  |  |  |
| 3.BREQ | 50.58 ± 10.57 | 0.452** | 0.249** | 1 |  |  |  |  |
| 4.GSES | 27.41 ± 7.11 | 0.539** | 0.480** | 0.472** | 1 |  |  |  |
| 5.PSSS | 53.40 ± 14.43 | 0.350** | 0.354** | 0.356** | 0.501** | 1 |  |  |
| 6.EAQ1 | 20.15 ± 4.54 | 0.935** | 0.281** | 0.453** | 0.524** | 0.354** | 1 |  |
| 7.EAQ2 | 7.59 ± 2.23 | 0.761** | 0.203** | 0.366** | 0.415** | 0.257** | 0.617** | 1 |
| 8.EAQ3 | 7.01 ± 2.58 | 0.763** | 0.210** | 0.272** | 0.370** | 0.227** | 0.573** | 0.383** |
| 9.SKQ1 | 2.85 ± 1.15 | 0.136** | 0.610** | 0.105* | 0.308** | 0.223** | 0.158** | 0.075 |
| 10.SKQ2 | 5.66 ± 1.79 | 0.212** | 0.623** | 0.179** | 0.279** | 0.180** | 0.231** | 0.145** |
| 11.SKQ3 | 7.88 ± 2.78 | 0.250** | 0.851** | 0.183** | 0.414** | 0.305** | 0.230** | 0.159** |
| 12.SKQ4 | 6.63 ± 2.36 | 0.189** | 0.798** | 0.205** | 0.331** | 0.278** | 0.171** | 0.156** |
| 13.SKQ5 | 2.17 ± 0.97 | 0.146** | 0.518** | 0.145** | 0.266** | 0.217** | 0.139** | 0.123** |
| 14.SKQ6 | 2.87 ± 1.09 | 0.220** | 0.552** | 0.196** | 0.365** | 0.226** | 0.221** | 0.167** |
| 15.BREQ1 | 6.71 ± 2.41 | 0.163** | 0.035 | 0.316** | 0.127** | 0.108* | 0.144** | 0.110* |
| 16.BREQ2 | 8.76 ± 3.12 | 0.301** | 0.243** | 0.646** | 0.335** | 0.272** | 0.278** | 0.290** |
| 17.BREQ3 | 8.06 ± 2.94 | 0.270** | 0.116** | 0.490** | 0.201** | 0.120** | 0.277** | 0.188** |
| 18,BREQ4 | 8.93 ± 2.45 | 0.359** | 0.218** | 0.858** | 0.416** | 0.298** | 0.380** | 0.294** |
| 19.BREQ5 | 8.97 ± 2.46 | 0.352** | 0.177** | 0.868** | 0.410** | 0.324** | 0.368** | 0.279** |
| 20.BREQ6 | 9.12 ± 2.52 | 0.361** | 0.198** | 0.837** | 0.408** | 0.312** | 0.374** | 0.292** |
| 21.PSSS1 | 17.58 ± 6.05 | 0.234** | 0.299** | 0.298** | 0.396** | 0.785** | 0.238** | 0.176** |
| 22.PSSS2 | 17.90 ± 6.08 | 0.287** | 0.277** | 0.272** | 0.359** | 0.793** | 0.296** | 0.200** |
| 23.PSSS3 | 17.87 ± 6.14 | 0.320** | 0.278** | 0.288** | 0.445** | 0.796** | 0.319** | 0.237** |
|  | 8 | 9 | 10 | 11 | 12 | 13 | 14 | 15 |
| 1.EAQ |  |  |  |  |  |  |  |  |
| 2.SKQ |  |  |  |  |  |  |  |  |
| 3.BREQ |  |  |  |  |  |  |  |  |
| 4.GSES |  |  |  |  |  |  |  |  |
| 5.PSSS |  |  |  |  |  |  |  |  |
| 6.EAQ1 |  |  |  |  |  |  |  |  |
| 7.EAQ2 |  |  |  |  |  |  |  |  |
| 8.EAQ3 | 1 |  |  |  |  |  |  |  |
| 9.SKQ1 | 0.074 | 1 |  |  |  |  |  |  |
| 10.SKQ2 | 0.118** | 0.355** | 1 |  |  |  |  |  |
| 11.SKQ3 | 0.225** | 0.401** | 0.382** | 1 |  |  |  |  |
| 12.SKQ4 | 0.144** | 0.401** | 0.290** | 0.598** | 1 |  |  |  |
| 13.SKQ5 | 0.096* | 0.218** | 0.262** | 0.382** | 0.314** | 1 |  |  |
| 14.SKQ6 | 0.140** | 0.287** | 0.245** | 0.365** | 0.378** | 0.204** | 1 |  |
| 15.BREQ1 | 0.151** | -0.097* | -0.013 | 0.058 | 0.033 | 0.029 | 0.108* | 1 |
| 16.BREQ2 | 0.181** | 0.103* | 0.195** | 0.163** | 0.199** | 0.164** | 0.178** | 0.077 |
| 17.BREQ3 | 0.177** | 0.099* | 0.063 | 0.097* | 0.085* | 0.044 | 0.089* | 0.044 |
| 18.BREQ4 | 0.179** | 0.116** | 0.179** | 0.156** | 0.170** | 0.117** | 0.146** | 0.066 |
| 19.BREQ5 | 0.190** | 0.086* | 0.136** | 0.125** | 0.139** | 0.113** | 0.129** | 0.093* |
| 20.BREQ6 | 0.197** | 0.099* | 0.141** | 0.137** | 0.172** | 0.121** | 0.141** | 0.067 |
| 21.PSSS1 | 0.147** | 0.190** | 0.165** | 0.246** | 0.243** | 0.179** | 0.179** | 0.056 |
| 22.PSSS2 | 0.187** | 0.129** | 0.138** | 0.244** | 0.218** | 0.206** | 0.184** | 0.114** |
| 23.PSSS3 | 0.214** | 0.216** | 0.129** | 0.246** | 0.213** | 0.138** | 0.177** | 0.088* |
|  | 16 | 17 | 18 | 19 | 20 | 21 | 22 | 23 |
| 1.EAQ |  |  |  |  |  |  |  |  |
| 2.SKQ |  |  |  |  |  |  |  |  |
| 3.BREQ |  |  |  |  |  |  |  |  |
| 4.GSES |  |  |  |  |  |  |  |  |
| 5.PSSS |  |  |  |  |  |  |  |  |
| 6.EAQ1 |  |  |  |  |  |  |  |  |
| 7.EAQ2 |  |  |  |  |  |  |  |  |
| 8.EAQ3 |  |  |  |  |  |  |  |  |
| 9.SKQ1 |  |  |  |  |  |  |  |  |
| 10.SKQ2 |  |  |  |  |  |  |  |  |
| 11.SKQ3 |  |  |  |  |  |  |  |  |
| 12.SKQ4 |  |  |  |  |  |  |  |  |
| 13.SKQ5 |  |  |  |  |  |  |  |  |
| 14.SKQ6 |  |  |  |  |  |  |  |  |
| 15.BREQ1 |  |  |  |  |  |  |  |  |
| 16.BREQ2 | 1 |  |  |  |  |  |  |  |
| 17.BREQ3 | 0.135** | 1 |  |  |  |  |  |  |
| 18.BREQ4 | 0.433** | 0.239** | 1 |  |  |  |  |  |
| 19.BREQ5 | 0.422** | 0.268** | 0.891** | 1 |  |  |  |  |
| 20.BREQ6 | 0.411** | 0.192** | 0.885** | 0.881** | 1 |  |  |  |
| 21.PSSS1 | 0.228** | 0.120** | 0.247** | 0.286** | 0.251** | 1 |  |  |
| 22.PSSS2 | 0.225** | 0.091* | 0.211** | 0.224** | 0.229** | 0.439** | 1 |  |
| 23.PSSS3 | 0.196** | 0.086* | 0.263** | 0.268** | 0.270** | 0.438** | 0.453** | 1 |

**Supplementary Table 3** The model fit of the IMB model

| Index | Criterion | IMB |
| --- | --- | --- |
| χ^2^/df | < 3.00 | 2.675 |
| RMSEA | < 0.08 | 0.055 |
| NFI | > 0.90 | 0.922 |
| IFI | > 0.90 | 0.950 |
| CFI | > 0.90 | 0.949 |
| GFI | > 0.90 | 0.975 |
| AGFI | > 0.90 | 0.952 |

**Supplementary Table 4** Path coefficients of the IMB model

| Path | Estimate | Std, Estimate | S.E. | P value |
| --- | --- | --- | --- | --- |
| Information ↔ Exercise adherence | 18.862 | 0.249 | 3.334 | <0.001 |
| Information ↔ Social support | 36.627 | 0.354 | 4.686 | <0.001 |
| Exercise adherence ↔ Social support | 54.243 | 0.356 | 6.905 | <0.001 |
| Information → Self-efficacy | 0.301 | 0.304 | 0.035 | <0.001 |
| Personal motivation → Self-efficacy | 0.197 | 0.293 | 0.023 | <0.001 |
| Social support → Self-efficacy | 0.142 | 0.289 | 0.018 | <0.001 |
| Personal motivation → Exercise adherence | 0.182 | 0.252 | 0.028 | <0.001 |
| Self-efficacy → Exercise adherence | 0.393 | 0.367 | 0.041 | <0.001 |

Std. Estimate standardized estimate, S.E. standard error

P value < 0.05 was considered significant
